# Osteoradionecrosis of the hyoid bone after intra-arterial chemoradiotherapy for oropharyngeal cancer: MR imaging findings

**DOI:** 10.1186/s40644-017-0123-7

**Published:** 2017-07-27

**Authors:** Hiromitsu Hatakeyama, Noriyuki Fujima, Kazuhiko Tsuchiya, Kenji Mizoguchi, Takatsugu Mizumachi, Tomohiro Sakashita, Satoshi Kano, Akihiro Homma, Satoshi Fukuda

**Affiliations:** 1 Department of Otolaryngology-Head and Neck Surgery, Hokkaido University Graduate School of Medicine, Kita 15, Nishi 7, Kita-ku, Sapporo, 060-8638 Japan; 2 Department of Radiology, Hokkaido University Graduate School of Medicine, Sapporo, Japan; 3 Department of Radiotherapy, Hokkaido University Graduate School of Medicine, Sapporo, Japan; 4 Department of Otolaryngology, Yokohama City University Graduate School of Medicine, Yokohama, Japan

**Keywords:** Oropharyngeal cancer, Hyoid bone, Osteoradionecrosis, Intra-arterial chemoradiotherapy, MR imaging

## Abstract

**Background:**

Osteoradionecrosis (ORN) of the hyoid bone sometimes induces severe front neck infection and can cause laryngeal stenosis and carotid rupture. Although ORN of the hyoid bone is known to be a complication of chemoradiotherapy for head and neck cancer, there has been no basis for its evaluation. Our purpose is to present the clinical and MR imaging features of ORN of the hyoid bone.

**Methods:**

The study group comprised patients with advanced oropharyngeal cancer treated with targeted intra-arterial cisplatin infusion with concomitant radiotherapy. ORN of the hyoid bone was identified on the basis of decreased signal intensity of the bone marrow on T1WI images. Signal intensity on T2WI images was used to distinguish between inflammation and fibrosis.

**Results:**

A total of 39 pre-treatment MR images and follow-up MR images were reviewed. ORN of the hyoid bone were detected in 30% of patients after treatment, with 23% of them showing inflammation and 7.7% fibrosis. Two patients developed severe neck infection and received antibiotics and underwent surgical intervention by tracheostomy and resection of the hyoid bone.

**Conclusion:**

Our MR imaging study showed that ORN of the hyoid bone is not particularly rare in patients with oropharyngeal cancer treated with chemoradiotherapy. Clinicians should evaluate images carefully to prevent the development of severe complication due to infection associated with ORN of the hyoid bone.

## Background

Osteoradionecrosis (ORN) of the hyoid bone is an infrequent and less critical complication, as compared with that of the jaw and thyroid cartilages, associated with chemoradiation treatment for oropharyngeal and laryngeal cancer [1, 2]. However, ORN of the hyoid bone sometimes induces severe front neck infection, laryngeal stenosis and carotid rapture [3, 4]. Most reports of ORN of the hyoid bone involve small clinical cases, and there has been only one report of a series of CT image studies [5].

Targeted intra-arterial cisplatin infusion with concomitant radiotherapy (hereafter called RADPLAT) is a useful modality for the treatment of head and neck cancer, particularly maxillary and base of tongue cancer [6–8]. RADPLAT has made it possible to administer high doses of cisplatin to the tumor lesion without severe systemic complications [9]. However, the high focal treatment intensity appears to lead to acute and late local complications, and the frequency of ORN of the hyoid may be higher than that observed in association with other therapies.

MR imaging is the best modality for the diagnosis of bone marrow disease, and MR imaging for the evaluation of osteonecrosis has been frequently and thoroughly reported [10–12]. Bone marrow necrosis is usually detected as areas of decreased signal on T1-weighted images [13], and we estimated the incidence of ORN of the hyoid based on MR imaging findings before and after RADPLAT treatment.

## Methods

### Patients

The study group comprised 39 Japanese patients who were diagnosed with oropharyngeal cancer and treated at Hokkaido University Hospital, Japan, between 2003 and 2014. The main clinical characteristics of the patients are shown in Table 1. The subjects included 31 men and 8 women with a median age of 60 years (range, 26–77 years). Demographic and clinicopathological data, including age, gender, smoking history, tumor stage, and clinical outcomes, were obtained from the patients’ charts. All patients were treated with RADPLAT. This study was approved by the Institutional Review Board of the Hokkaido University Hospital.

**Table 1:** Clinicopathological feature of patients (*n* = 39)

Age(years old)		26–77
		(median = 60)
Gender	Male	31
	Female	8
Stage	II	2
	III	4
	IV	33
Primary Site		
Anterior Wall		34
Lateral Wall		5
Histology		
SCC		33
ACC		6

SCC: Squamous Cell Carcinoma

ACC: Adenoid Cystic Carcinoma

### Chemotherapy

All patients received concurrent intra-arterial cisplatin and intravenous sodium thiosulfate infusions as follows: cisplatin (100–120 mg/m2 per week for four weeks) was infused through a microcatheter placed angiographically to selectively encompass only the dominant blood supply of the targeted tumor. Tumors of the anterior wall of the oropharynx are usually supplied by the lingual artery, but in cases where the facial artery or the superior thyroid artery supplied the tumor, part of the dose was administered through these alternative arteries. Simultaneously, sodium thiosulfate (20–24 g) was given intravenously, as described by Robbins, to neutralize the cisplatin [9]. Chemotherapy was completed during the first four weeks provided that patients responded well in the early treatment period and received three arterial infusions.

### Radiotherapy

All patients were treated with external beam therapy without brachytherapy. Until May 2006, the irradiation schedule was 65Gy in 26 fractions or 66 Gy in 30 fractions over 6.5 weeks. Since that time, 70 Gy has been given in 35 fractions over seven weeks for all patients with advanced head and neck cancer. After administration of the initial dose of 40–44 Gy, an additional 22–30 Gy was applied to a more shrunken field, focusing on the primary tumor bed and the positive lymph nodes. Both the initial and shrunken fields included the hyoid bone area. The dose to the spinal cord was kept below 40 Gy in all instances.

### MR imaging

All MR imaging was performed using either 1) a 3.0-Tesla unit (Achieva TX; Philips Healthcare, Bests, the Netherlands) with a 16-channel neurovascular coil or 2) a 1.5-Tesla unit (MAGNETOM Symphony; Siemens, Erlangen, Germany) with a neck matrix coil. The MR images included both axial T1-weighted (T1WI) and T2-weighted images (T2WI). A spin-echo sequence was used for T1WI and a turbo-spin echo (TSE) sequence with fat suppression of spectral attenuated inversion recovery (SPAIR) technique was used for T2WI.The imaging parameters for T1WI were as follow: 1) (Philips scanner) TR 450 msec, TE 10 msec, FOV 240 × 240 mm, 512 × 512 matrix, slice thickness 5 mm, and interslice gap 30%; and 2) (Siemens scanner) TR 600 msec, TE 12 msec, FOV 240 × 240 mm, 320 × 320 matrix, slice thickness 5 mm, and interslice gap 50%. The imaging parameters for T2WI were as follow: 1) (Philips scanner) TR 4500 msec, TE 70 msec, TSE factor 9, FOV 240 × 240 mm, 512 × 512 matrix, slice thickness 5 mm, and interslice gap 30%; and 2) (Siemens scanner) TR 4780 msec, TE 92 msec, TSE factor 9, FOV 220 × 180 mm, 380 × 310 matrix, slice thickness 5 mm, and interslice gap 50%.

### MR image evaluation

The T1WI and T2WI were visually evaluated. ORN of the hyoid bone was identified as regions within the hyoid bones with decreased bone marrow signal intensity on the T1WI. In addition, a high signal intensity on the T2WI was considered to represent inflammation or necrotic tissue caused by ORN. MR image interpretation was performed using XTREK VIEW software (J-MAC system, Inc., Tokyo, Japan). All images were reviewed by two raters with consensus reading; a board-certified neuroradiologist with 11 years of experience and a board-certified otolaryngologist with 13 years of experience in otolaryngology and 5 years experience in head and neck surgery (N.F. and H.H.).

### [18F] fluoride PET/CT evaluation

Evaluation of [18F] fluoride PET/CT images was also performed on XTREK VIEW software (J-MAC system, Inc., Tokyo, Japan).

The extent of pathologically high tracer uptake of the hyoid bone as visualized with PET was anatomically assigned using the coregistered CT and noted on the hyoid bone scheme. Fluoride uptake had to be at least 2 times higher than physiologic uptake of healthy appearing bone. Maximum standardized uptake values, corrected for body weight, were used to measure [18F] fluoride uptake.

## Results

**Fig. 1:**
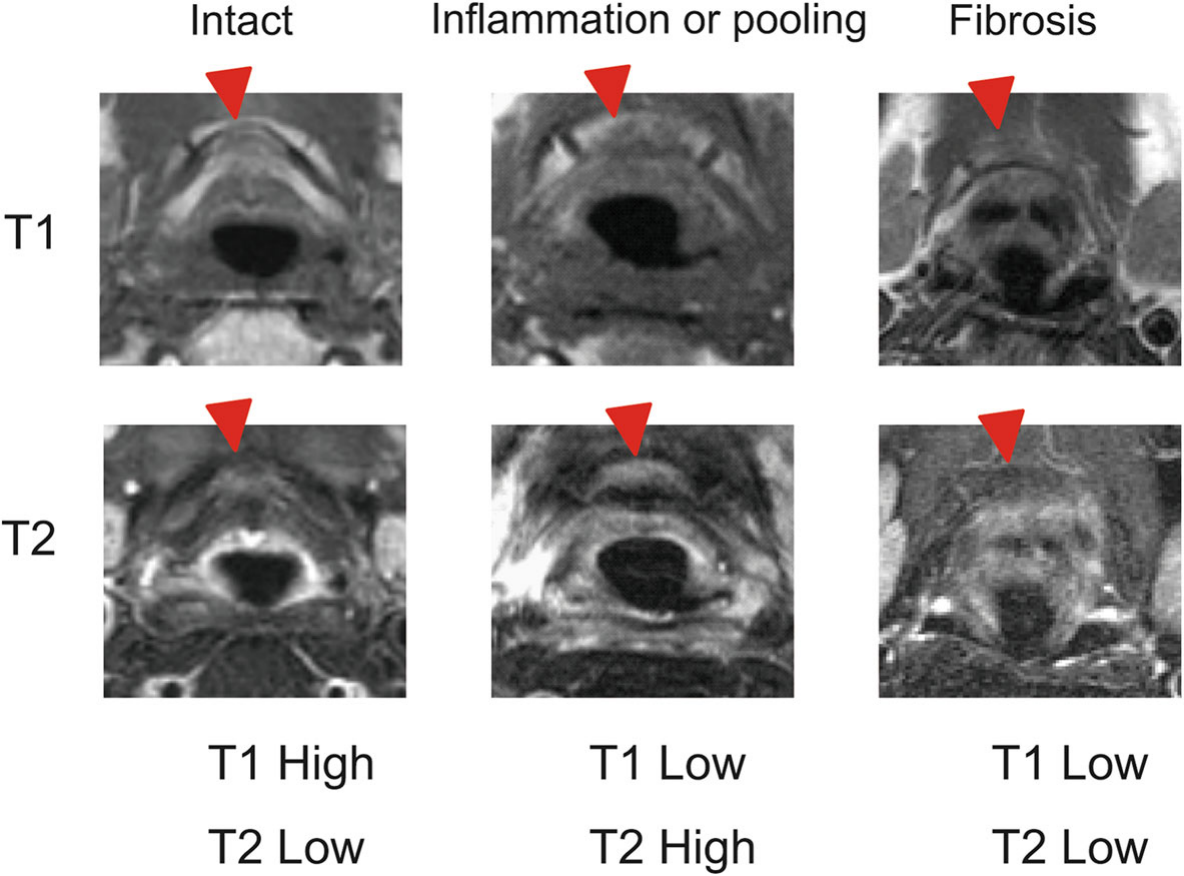
Representative MR images of the hyoid bone after treatment. All images were categorized into three groups; the intact, inflammation and fibrosis groups

Clinical data from 39 patients are shown in Table 1. All patients received a full-course of radiotherapy without interruption. Five patients received a total of 65 Gy of radiation, 8 patients received a total of 66 Gy of radiation, and the other 26 patients received 70 Gy. Fourteen patients received 3 times weekly intra-arterial chemotherapy, and 25 patients received 4 times weekly intra-arterial chemotherapy. We reviewed a total of 39 pre-treatment and follow-up MR images. The elapsed time between final irradiation and follow-up MR imaging for this review ranged between 3 and 14 months.(median: 6 month and mean: 5.3 month) The hyoid bone marrow of all 39 patients showed high signals on T1WI and low signals on T2WI in the pre-treatment MR images. We categorized MR images of the hyoid bone into 3 patterns depending on the signal intensities on T1WI and T2WI. Representative MR images of the intact hyoid bone show a high signal on T1WI in the bone marrow and a decreased signal on T2WI [12]. MR images of the hyoid with a decreased signal on T1WI and high signal on T2WI were categorized into the inflammation group and MR images of the hyoid with low signals on both T1WI and T2WI were categorized into the fibrous change group (Fig. 1). A high signal on T1WI and low signal on T2WI in the bone marrow were observed in 27 cases post-treatment.A partial or total low signal on T1WI in the body of hyoid bone was observed in 12 cases. No decreased signals were observed at the horn of the hyoid on T1WI. Of the 12 cases, 9 cases showed a high signal and 3 cases showed a decreased signal on T2WI (Table 2). Median periods from the final treatment to the scan of intact, inflammation and fibrosis group is 6, 6 and 5 month, respectively. Mean periods of them of intact, inflammation and fibrosis group is 5.8, 4.8 and 5 months. No significant difference in median and mean periods for MR imaging among 3 groups.

**Table 2:** Signal intensity of the hyoid bone in the post-treatment MR images

		T2WI	
		High	Low
T1WI	High	0 (0%)	27 (69.2%)
	Low	9 (23%)	3 (7.7%)

### Severe front neck infections

**Fig. 2:**
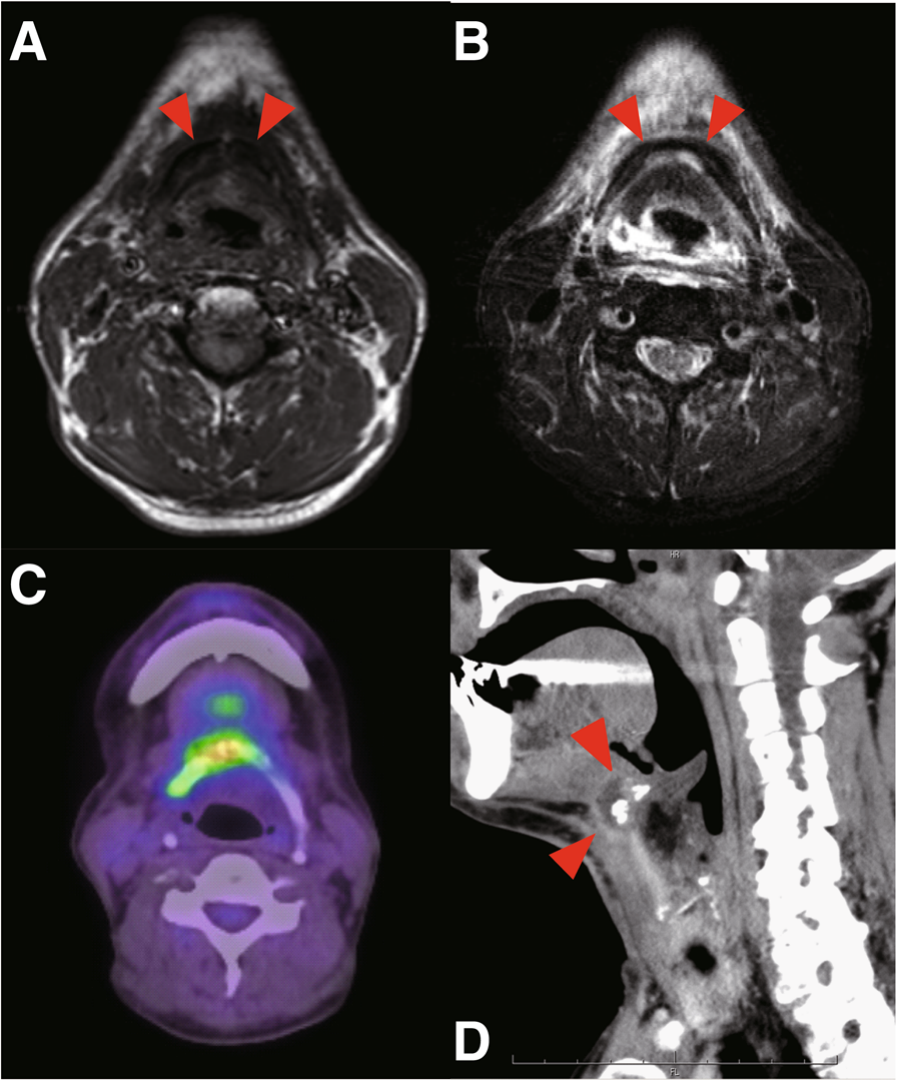
**a** Signal intensity partially decreased in the hyoid on T1WI, **b** Mixed high and low signal intensity on T2WI The arrows indicate the ORN of hyoid bone. **c** The PET-CT image: High FDG activity is observed in the hyoid. **d** Sagittal view of the CT scan image in the larynx. The *red arrows* indicate disruption of the hyoid with abscess formation

**Fig. 3:**
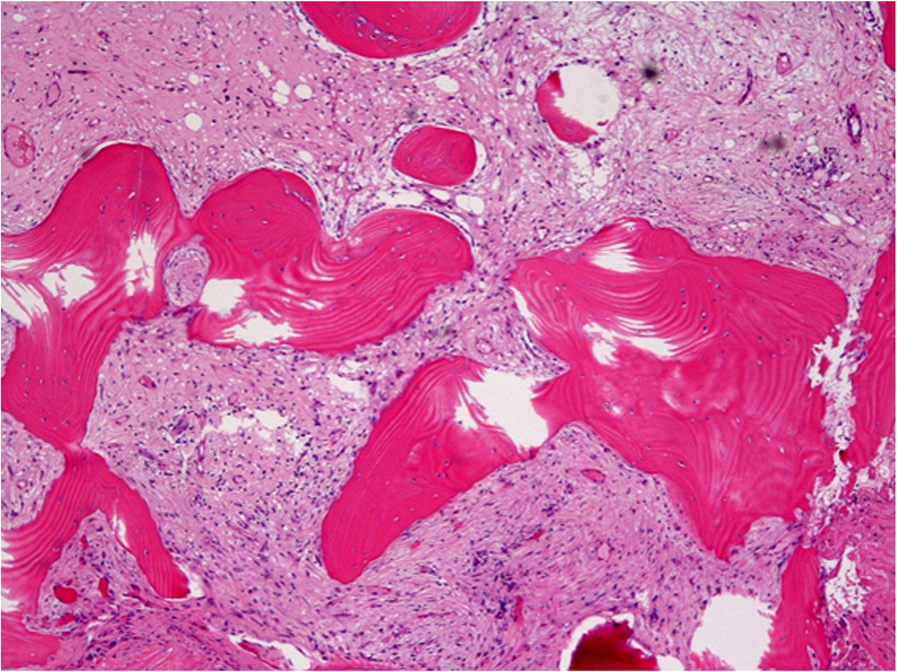
Histopathological image of the resected hyoid bone which with cortical disruption, and surrounding air and abscess formation

Two patients had severe front neck infection caused by ORN of the hyoid bone. The first case was a 64-year-old male with primary ulcerative squamous cell carcinoma at the base of the tongue. He experienced repeated bleeding from the ulcerative lesion tumor, and embolization of the lingual artery was performed at 1 week before the first radiotherapy. He received 70Gy of radiotherapy in 35 fractions and intra-arterial infusion of 100 mg/m2 of cisplatin (4×). No severe acute complications were observed during RADPLAT. After the treatment, his primary tumor disappeared and he was diagnosed as a complete response. However, 6 months after the radiotherapy, he experienced front a neck infection with swelling of the oropharynx and larynx. MR imaging showed a low signal intensity at the hyoid bone on T1WI and a mixed low and high signal at the hyoid on T2WI (Fig. 2ab). We treated the patient with antibiotics and hyperbaric O2 therapy and the infection was resolved; however, repeat infections were observed a total of 3 times within a 6-month period. PET-CT imaging was undertaken at 12 months after the irradiation, demonstrating focally high FDG activity at the right hyoid bone (SUV = 13.9; Fig. 2c). Cortical disruption and air and abscess formation surrounding the hyoid were observed (Fig. 2d). For pathological diagnosis and prevention of further infections, the hyoid bone with the surrounding tissue was resected and the pharyngeal fistula was covered with a portion of the right sternal branch of the sternocleido muscle. Histopathological analysis of the resected samples showed no malignant lesion in the hyoid bone or pharyngeal mucosa. However, the resected hyoid bone showed necrotic and fibrous changes surrounding an area of invasion by lymphocytes, histiocytes, plasma and neutrophils (Fig. 3).

**Fig. 4:**
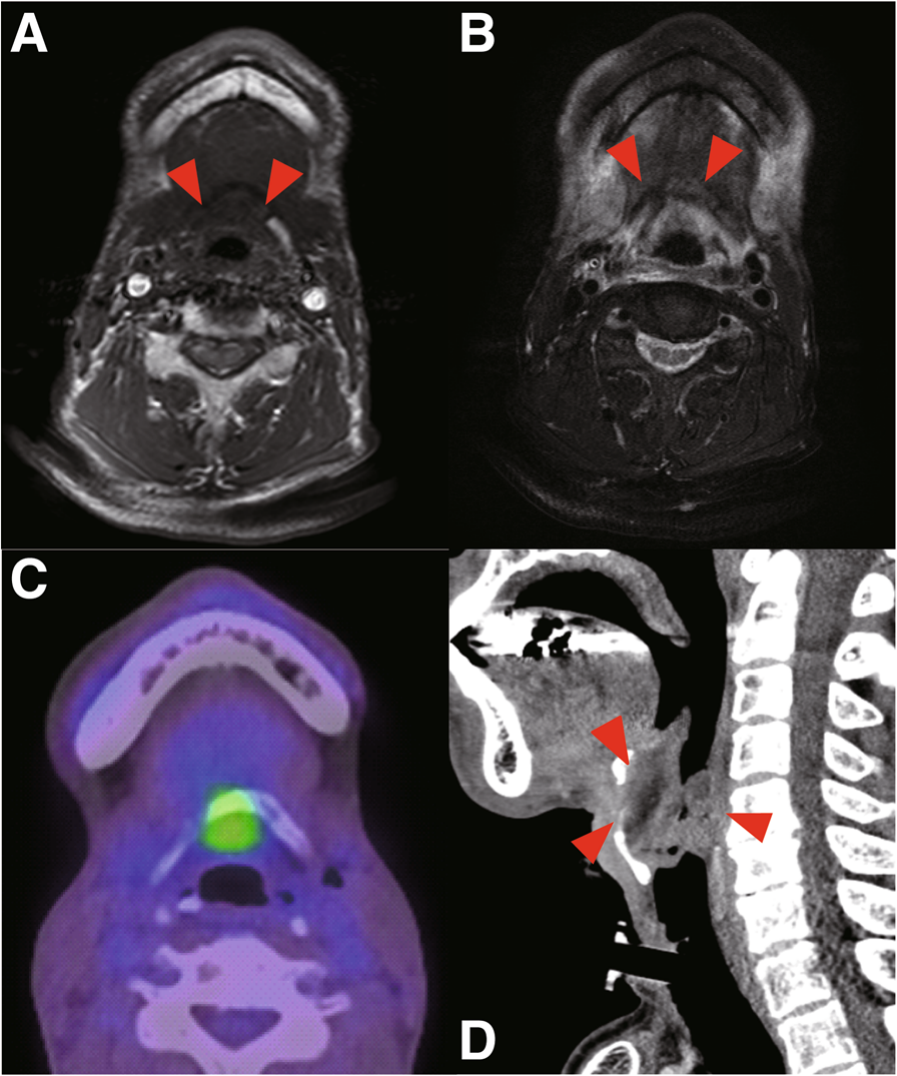
**a** Signal intensity decreased in the hyoid on T1WI, **b** Mixed high and low signal intensity on T2WI The *arrows* indicate the ORN of hyoid bone. **c** PET-CT shows focal high FDG activity in the hyoid and larynx. **d** Sagittal view of CT scan in the larynx. The *red arrows* indicated edematous swelling of laryngeal mucosa and stenosis

The second case was a 60-year-old male with base of tongue adenoid cystic carcinoma, T4N1M0. He received 70Gy of radiotherapy and intra-arterial infusion of 100 mg/m2 of cisplatin (4×), and achieved a complete response. At six months after the last irradiation, laryngeal stenosis caused by edematous swelling of the larynx and infection of the hyoid were observed. MR imaging showed a low signal intensity at the hyoid bone body and right horn on T1WI and a high signal at the hyoid bone body on T2WI (Fig. 4ab). Increased FDG activity was observed at the hyoid bone (SUV = 6.6) and this lesion was suspected of indicating recurrence or inflammation (Fig. 4cd). Emergency tracheostomy, incisional drainage and biopsy were performed. Ceftriaxone injections were administered for one week perioperatively, and then switched to oral clindamycin. The laryngeal stenosis was resolved and the tracheostomy was closed at 1 month after the surgical treatment. We treated with long-term clindamycin and no recurrence of infection was observed for 2 years.

## Discussion

In most previous case reports of ORN of the hyoid bone, the condition was diagnosed on the basis of CT scans, not MR images, [5, 14, 15] although Smith et al. reported one case diagnosed by MR imaging [3]. In most of our cases, follow-up MR imaging was performed as MR images are more useful for the visualization of primary tumors at base of tongue than enhanced CT images. Further, as most elderly Japanese patients have metal-based dental restoration work, such as dental crowns, metal streak artifacts are commonly observed in CT scans of the pharynx and oral cavity. These metal-based restoration materials produce fewer artifacts on MR images [16].

CT scan images can reveal ORN of the hyoid bone involving cortical disruption, pathologic fracture or fragmentation, exposure to the pharynx and air within or surrounding the hyoid [5]. However, it is difficult to detect fibrous changes in the bone marrow by CT scan. MR imaging of osteonecrosis of the mandible and femoral head has been frequently reported and commonly used for diagnostic purposes by radiologists [11–13]. The hyoid bone contains a relatively small amount of bone mass comparing with the jaws or other bone, and a few slice image of the hyoid could be estimated. But MR images were found to be useful in the evaluation of the hyoid bone marrow in our study. A high signal on T2-weighted images was interpreted as indicative of the presence of inflammation or fluid and might be predictive of severe infection. Fibrous changes in the hyoid bone are difficult to detect using CT scans, but could be detected as areas of low signal in the bone marrow on both T1- and T2-weighted MR images.

MR imaging was able to detect ORN of the hyoid bone in 30% patients with oropharyngeal cancer treated with RADPLAT. Although we could not find any previous reports on the rate of ORN of the hyoid, this rate appears rather high. The cause of this high rate of ORN might be the higher regional therapeutic intensity of RADPLAT and detection sensitivity of MR imaging. Eighty-three percent of cases of ORN of the hyoid consisted of fibrous changes and did not show any clinical symptoms, such as trisimus, dysphagia or throat pain. However, 2 cases developed severe frontal neck infection and these were cured with surgical intervention involving tracheostomy and the resection of hyoid bone and antibiotics. Patients with potential hyoid bone fibrosis should be observed without treatment, although some kind of preventive treatment is desirable for patients expected of severe inflammation、Hyperbaric O2 therapy is commonly used for ORN, and we applied it in the treatment of Case 1 [17]. Combination therapy involving antibiotics and hyperbaric O2 was able to resolve the inflammation temporarily, but it relapsed soon after treatment. Regulation of the infection following ORN of the mandible was achieved by long-term treatment with antibiotics [18]. Though resection of the fibrous hyoid bone can induces a pharyngocutaneous fistula, long-term antibiotics treatment is a favorable treatment option for the prevention of severe infection if ORN of the hyoid is detected after chemoradiotherapy.

## Conclusion

The results of this MR imaging study suggested that ORN of the hyoid bone is not particularly rare in patients with oropharyngeal cancer treated with RADPLAT. Most of patients with hyoid fibrosis did not show clinical symptoms, although the ORN was sometimes accompanied by severe neck infection. MR imaging is commonly used for observation after chemoradiotherapy in the patients with oropharyngeal cancer and is useful in the diagnosis of ORN. Clinicians should evaluate images carefully for findings indicative of ORN of the hyoid bone during follow-up after treatment.

## Abbreviations

ORN: Osteoradionecrosis
RADPLAT: Radiation therapy and targeted cisplatin chemotherapy

## References

[1] Lambade PN, Lambade D, Goel M (2013). Osteoradionecrosis of the mandible: a review. Oral Maxillofac Surg.

[2] Chen JA, Wang CC, Wong YK, Wang CP, Jiang RS, Lin JC, Chen CC, Liu SA. Osteoradionecrosis of mandible bone in patients with oral cancer - associated factors and treatment outcomes. Head Neck. 2016;38(5):762-8. 10.1002/hed.23949 25521838

[3] Smith WK, Pfleiderer AG, Millet B (2003). Osteoradionecrosis of the hyoid presenting as a cause of intractable neck pain following radiotherapy and the role of magnetic resonance image scanning to aid diagnosis. J Laryngol Otol.

[4] Chard R, Monroe MM, Andersen PE (2013). Hyoid osteoradionecrosis associated with carotid rupture: report of 2 cases. Head Neck.

[5] Yoo JS, Rosenthal DI, Mitchell K, Ginsberg LE (2010). Osteoradionecrosis of the hyoid bone: imaging findings. AJNR Am J Neuroradiol.

[6] Kano S, Homma A, Oridate N, Suzuki F, Hatakeyama H, Mizumachi T, Furusawa J, Sakashita T, Yoshida D, Onimaru R (2011). Superselective arterial cisplatin infusion with concomitant radiation therapy for base of tongue cancer. Oral Oncol.

[7] Homma A, Sakashita T, Yoshida D, Onimaru R, Tsuchiya K, Suzuki F, Yasuda K, Hatakeyama H, Furusawa J, Mizumachi T (2013). Superselective intra-arterial cisplatin infusion and concomitant radiotherapy for maxillary sinus cancer. Br J Cancer.

[8] Homma A, Oridate N, Suzuki F, Taki S, Asano T, Yoshida D, Onimaru R, Nishioka T, Shirato H, Fukuda S (2009). Superselective high-dose cisplatin infusion with concomitant radiotherapy in patients with advanced cancer of the nasal cavity and paranasal sinuses: a single institution experience. Cancer.

[9] Robbins KT, Kumar P, Wong FS, Hartsell WF, Flick P, Palmer R, Weir AB, Neill HB, Murry T, Ferguson R (2000). Targeted chemoradiation for advanced head and neck cancer: analysis of 213 patients. Head Neck.

[10] Vogler JB, Murphy WA (1988). Bone marrow imaging. Radiology.

[11] Deshpande SS, Thakur MH, Dholam K, Mahajan A, Arya S, Juvekar S (2015). Osteoradionecrosis of the mandible: through a radiologist's eyes. Clin Radiol.

[12] Hu LB, Huang ZG, Wei HY, Wang W, Ren A, Xu YY (2015). Osteonecrosis of the femoral head: using CT, MRI and gross specimen to characterize the location, shape and size of the lesion. Br J Radiol.

[13] Stevens SK, Moore SG, Kaplan ID (1990). Early and late bone-marrow changes after irradiation: MR evaluation. AJR Am J Roentgenol.

[14] Gill AS, Joshi AS: Osteoradionecrosis of the hyoid bone - a novel application of the Sonopet ultrasonic aspirator. BMJ Case Rep. 2014;2014. 10.1136/bcr-2014-205682 PMC4173191 25246467

[15] Monceaux G, Perie S, Montravers F, Angelard B, Corlieu P, St Guily JL (1999). Osteoradionecrosis of the hyoid bone: a report of 3 cases. Am J Otolaryngol.

[16] Klinke T, Daboul A, Maron J, Gredes T, Puls R, Jaghsi A, Biffar R (2012). Artifacts in magnetic resonance imaging and computed tomography caused by dental materials. PLoS One.

[17] Schwartz HC (1999). Osteoradionecrosis and hyperbaric oxygen. Br J Oral Maxillofac Surg.

[18] Ruggiero S, Gralow J, Marx RE, Hoff AO, Schubert MM, Huryn JM, Toth B, Damato K, Valero V (2006). Practical guidelines for the prevention, diagnosis, and treatment of osteonecrosis of the jaw in patients with cancer. J Oncol Pract.

